# The Code of Ethics

**Published:** 1903-03

**Authors:** 


### The Code of Ethics.

IN A very practical and sensible commentary upon the code of medical ethics, the *Mississippi Medical Record*, February, 1903, says:

Professional Ethics vs. Commercialism is the text from which Dr. Gillmore, of Chicago, in the *Journal of the American Medical Association*, January 3, preaches a discourse that cannot but be depressing to the young men into whose hands it may fall. The doctor tells of one or two instances in which he has been treated in a most unethical manner by professional brethren, touches on irregular methods employed by physicians with more ambition than sense of decency and concludes with the suggestion that medical students shall be educated in ethics while at college.

When our code of ethics was written the country was controlled by an aristocracy. Say what they might about all men being born "free and equal," and no greater falsehood was ever put in print, there was then an aristocracy of wealth and learning that controlled affairs far more than is the case today. The learned professions as they were called, "Law, Medicine and Divinity," were in the hands of this aristocracy and our code is one of the greatest proofs of this fact. It necessarily follows that it cannot serve to meet the conditions we now face. Indeed, it is doubtful if any code will completely answer all requirements, for while the physician of gentlemanly instincts needs no written law to direct him in keeping his hands clean, the boor will ride roughshod over everything that lies between himself and the almighty dollar. As an example: the writer knows a physician who has probably not seen a copy of the code in a quarter of a century, and yet it is a positive pleasure to note the careful courtesy with which he treats his consultants and the high moral stand he takes in the conduct of his professional affairs—he is a gentleman by instinct. On the other hand we can cite an instance of a man whose mouth fairly reeks with quotations from the code and who never misses an opportunity to decry his brother physicians. There are probably few of us who cannot furnish similar illustrations from personal experience.

The *Record* does not think that the method advocated by Dr. Gillmore will prove an adequate remedy for the evil complained. Continuing the editorial says:

We believe we must get nearer the root of the matter. By insisting on a higher standard of educational requirements for entrance to our medical colleges we will get a better class of men—men who are not only from such environment as instils into them a sense of decency, but also who, being better prepared, can become better equipped mentally and will not be so readily tempted from ethical paths.

In the last paragraph quoted the *Record* makes an important suggestion of relief, which will do much toward meeting the difficulty. When the doctrine of state control is accepted and carried out loyally by medical schools, commonwealths and the profession in general, the question of medical ethics will be met as far as it can be by any written law or rule.

It is through education that ethical progress must be made and not through edicts pronounced, *ex cathedra*, with a top-lofty flourish or trumpet-like blare. A uniformity in standards of requirements for admission to medical schools, a similarity in length of terms and teaching methods, and a final examination by the state is the trinity that will overcome ethical paradoxes and establish reciprocity in licensure.

